# Pioglitazone Improves Reversal Learning and Exerts Mixed Cerebrovascular Effects in a Mouse Model of Alzheimer’s Disease with Combined Amyloid-β and Cerebrovascular Pathology

**DOI:** 10.1371/journal.pone.0068612

**Published:** 2013-07-18

**Authors:** Panayiota Papadopoulos, Pedro Rosa-Neto, Joseph Rochford, Edith Hamel

**Affiliations:** 1 Laboratory of Cerebrovascular Research, Montreal Neurological Institute, McGill University, Montréal, Québec, Canada; 2 Brain Imaging Centre, Montreal Neurological Institute, McGill University, Montréal, Québec, Canada; 3 Douglas Hospital Research Centre, McGill University, Montréal, Québec, Canada; Nathan Kline Institute and New York University School of Medicine, United States of America

## Abstract

Animal models of Alzheimer’s disease (AD) are invaluable in dissecting the pathogenic mechanisms and assessing the efficacy of potential new therapies. Here, we used the peroxisome proliferator-activated receptor gamma agonist pioglitazone in an attempt to rescue the pathogenic phenotype in adult (12 months) and aged (>18 months) bitransgenic A/T mice that overexpress a mutated human amyloid precursor protein (APPSwe,Ind) and a constitutively active form of transforming growth factor-β1 (TGF-β1). A/T mice recapitulate the AD-related cognitive deficits, amyloid beta (Aβ) and cerebrovascular pathologies, as well as the altered metabolic and vascular coupling responses to increased neuronal activity. Pioglitazone normalized neurometabolic and neurovascular coupling responses to sensory stimulation, and reduced cortical astroglial and hippocampal microglial activation in both age groups. Spatial learning and memory deficits in the Morris water maze were not rescued by pioglitazone, but reversal learning was improved in the adult cohort notwithstanding a progressing Aβ pathology. While pioglitazone preserved the constitutive nitric oxide synthesis in the vessel wall, it unexpectedly failed to restore cerebrovascular reactivity in A/T mice and even exacerbated the dilatory deficits. These data demonstrate pioglitazone’s efficacy on selective AD hallmarks in a complex AD mouse model of comorbid amyloidosis and cerebrovascular pathology. They further suggest a potential benefit of pioglitazone in managing neuroinflammation, cerebral perfusion and glucose metabolism in AD patients devoid of cerebrovascular pathology.

## Introduction

Alzheimer’s disease (AD), the most common form of senile dementia in the elderly, is characterized by increased levels of amyloid-β (Aβ) peptide and neurofibrillary tangles, and by neuroinflammation. It is also marked by early decreases in cerebral glucose uptake (CGU) and cerebral blood flow (CBF), and by a cerebrovascular pathology [1]. The latter is multifaceted and involves a vascular fibrosis with thickening of the basement membrane, which has been imputed to increased levels of transforming growth factor-β1 (TGF-β1) [2–4]. Hence, in order to better recapitulate the complexity of the AD pathology, a double transgenic mouse model that concurrently overproduces Aβ and TGF-β1 (A/T mice) has been characterized [5]. A/T mice display the broad spectrum of cerebrovascular, neuronal, glial, and cognitive alterations found in AD patients, thereby integrating the comorbid factor of cerebrovascular pathology to that of increased amyloidosis in the pathogenesis of AD [6].

Pioglitazone is a peroxisome proliferator-activated receptor-γ (PPARγ) agonist that remains a potential candidate for AD therapy [7,8]. This interest was originally sparked by pioglitazone’s ability to cross the blood-brain-barrier (BBB) [9] and subsequent findings of its benefits against multiple features of AD pathology. In mice overexpressing mutated human amyloid precursor protein (APP mice), pioglitazone reduced glial inflammation, normalized CGU and cerebrovascular function, despite limited or no effect on Aβ processing and deposition [10–12]. Pioglitazone was similarly effective in restoring CGU and cerebrovascular function in mice that overexpress TGF-β1 [13,14], faithfully reproducing the AD cerebrovascular pathology [4]. However, further testing of pioglitazone therapy in AD might gain from a thorough examination of its efficacy in a more complex model, such as the A/T mice.

Hence, we investigated the effects of pioglitazone in adult and aged A/T mice on AD hallmarks including impaired neuronally-induced CGU and CBF responses, glial activation, amyloidosis and hippocampus-based learning and memory deficits. Cerebral arterial responsiveness and proteins regulating vascular structure or function were also evaluated. Despite aggravating effects of pioglitazone on vasodilatory responses, our findings support the use of pioglitazone as a strategy to delay the progression of the disease, particularly in AD patients free of vascular diseases, by demonstrating efficacy on AD-related neuroinflammation, hypoperfusion and hypometabolism together with benefits on cognitive flexibility.

## Methods

### Animals

A/T mice [5] overexpress a mutated form of the human amyloid precursor protein (APPSwe,Ind) driven by the platelet-derived growth factor β promoter (line J20) [15] and a constitutively active form of TGF-β1 under the control of the glial fibrillary acidic protein (GFAP) promoter on a C57BL/6J background (line T64) [4]. The expression of both transgenes was identified by touchdown PCR using tail-extracted DNA [5]. Adult (12 months old at endpoint; treated for 6 months, N=8-11/group) and aged (18-21 months old at endpoint; treated for 3 months N=5-7/group) A/T mice and wild-type (WT) littermates (body weight, ~30-60g) were treated or not with pioglitazone (20mg/kg/day in chow) with males and females evenly distributed in each group. Water and chow were available *ad libitum*. At the end of treatment, pioglitazone treated-A/T mice of both cohorts had gained more weight compared to age-matched WT or untreated A/T mice [adult (g): WT: 12.5 ± 1.0; WTpio: 8.6 ± 0.9; A/T: 11.7 ± 0.9; A/Tpio 21.6 ± 1.1; p<0.05; aged (g): WT: 2.5 ± 1.5; WTpio: 5.2 ± 2.3; A/T: 2.6 ± 0.5 vs A/Tpio 10.0 ± 0.9; p<0.05]. Fasting glycemia measured with a commercial glucometer (One Touch Ultra, LifeScan, Burnaby, BC, Canada) using blood collected from the mouse’s tail vein was not altered by treatment at any age [adult (mmol/L): WT 9.5 ± 0.4, WTpio 9.0 ± 0.3, A/T 8.7 ± 0.5, A/Tpio 8.8 ± 0.4; aged (mmol/L): WT 9.5 ± 0.5; WTpio 8.8 ± 0.3; A/T 9.2 ± 0.3; A/Tpio 8.4 ± 0.2]. All experiments were approved by the Animal Ethics Committee of the Montreal Neurological Institute, and abided by the guidelines of the Canadian Council on Animal Care.

**Morris Water Maze. (i) Spatial Learning and Memory**. Mice were trained to escape onto a platform located in a circular pool (1.4 m diameter) filled with opaque water (18±1°C) and located in a room with visual wall cues. The pool was subdivided into four quadrants numbered in a clockwise order – quadrant 1 being located north-east. There were two platform locations: (1) the visible platform (original quadrant; south-east quadrant 2) for a three-day training session, and (2) the hidden platform (target quadrant; north-west quadrant 4) submerged ~1cm below the surface of the water for a five-day training session. The location of the distal wall cues was randomly changed between the visible and hidden platform sessions, as previously described [16]. Mice were given three trials daily (spaced 45min apart) with a maximum duration of 60s/trial and 90s/trial for the visible and hidden platform training sessions, respectively. On the first day of each session, mice that failed to locate the platform in the allotted time were guided to and allowed to stay on it for 10s. Spatial memory was tested during the probe trial (60s, platform removed) 24h after the last hidden platform trial (day 9). Mice were kept warm with a heating lamp and testing began at the same time every day. Escape latencies and probe trial measures were recorded with the 2020 Plus tracking system and Water 2020 software (Ganz FC62D video camera; HVS Image, Buckingham, UK) [14]. The parameters of the probe trial were: percent time spent and distance traveled in target quadrant, average distance to platform location (the Gallagher measure [17]) and swim speed. The probe trial analysis was additionally subdivided in temporal patterns of search in the target quadrant presented in slices of 15s each [18]. 

**(ii) Cognitive flexibility**. The inability to switch to a new platform location while failing to suppress the execution of a previously learned task is a correlate of impaired cognitive flexibility [19,20]. To examine such possible alterations, the probe trial analysis was further extended to the assessment of the same abovementioned parameters in the original quadrant. Subsequent experiments resumed 2 days later.

### Cerebral glucose uptake (CGU)

This test was performed only in mice from the aged cohort. Mice were fasted for 12h before being scanned for CGU of 2-deoxy-2-[^18^F] fluoro-D-glucose ([^18^F] FDG) under isoflurane (1–2% in medical air) anesthesia in a CTI Concorde R4 micropositron emission tomography (microPET) scanner (Siemens Preclinical Solutions, Knoxville, TN, USA). Mice were injected with 100-150 µCi (100 µl) of [^18^F] FDG into the tail vein prior to a 45min uninterrupted unilateral (right side) whisker stimulation (8–10 Hz, electrical toothbrush) followed by a 25min PET acquisition (15min emission/10min transmission scan using a [^57^Co] point source). Physiological parameters (body temperature, cardiac rate and respiration) were constantly monitored (Biopac Systems, Goleta, CA, USA), and remained stable throughout the experiment. The reconstruction of the functional metabolic images was generated based on a maximum a posteriori probability algorithm for the [^18^F] FDG standard uptake values (SUV) adjusted according to body weight, injected dose of radioligand and fasting glycemia measurements taken before and after the scanning for all animals. Images were linearly coregistered (7 parameters) on strain-specific magnetic resonance imaging (MRI) templates generated on a 7-T Bruker Pharmascan system (Bruker Biospin, Ettlingen, Germany) from similarly aged WT and A/T mice (n = 5 per group) (for details [14,21]). Image analysis was conducted with a platform based on minc toll kit available online at: http://www.bic.mni.mcgill.ca/ServicesSoftware/HomePage. The areas of interest for this study were drawn on the somatosensory cortices [22]. Percentage activation was then defined as the metabolic index relative to the percentage of ([^18^F] FDG) SUV ratio between the maximally activated contralateral vs ipsilateral somatosensory cortex upon whisker stimulation. Final estimations were adjusted to animal body weight and injected radioactivity dose.

### Cerebral blood flow (CBF)

CBF increases induced by unilateral (right side) whisker stimulation were measured using laser Doppler flowmetry (Transonic Systems Inc, Ithaca, NY) two days following the Morris watermaze (adult mice) or PET session (aged mice). Mice were anesthetised with a mixture of ketamine (85 mg/kg, intramuscularly (i.m.); Bioniche, Belleville, ON) and xylazine (3 mg/kg i.m.; Haver, Etobicoke, ON), and fixed in a stereotaxic frame. The bone over the left barrel cortex was thinned to translucency for positioning of the laser probe, as previously described [14]. Body temperature was kept stable throughout the experiment with a heating pad. CBF was measured before, during and after whisker stimulation (4-5 stimulations, 8-10 Hz, 20s each interspaced by 1 min, electric toothbrush), and averaged for each mouse. Results are expressed as percentage increase of CBF from baseline. Experiments lasted ~20min/mouse, which allows for stable physiological parameters [21], and the experimenter was blind to the identity of the mouse.

### Tissue collection and preparation

At the end of the *in vivo* testing, a subset of mice from both cohorts was sacrificed by cervical dislocation for functional reactivity studies of the middle cerebral artery (MCA) (see below). The remaining vessels of the circle of Willis with their cortical branches and attached pial membrane, together with cortex and hippocampus from one hemibrain were removed, frozen on dry ice and stored (-80° C) for subsequent ELISA and Western blot experiments. The other hemisphere was immersion-fixed (4% paraformaldehyde (PFA) in 0.1 M phosphate buffer (PB), pH 7.4, overnight, 4° C), cryoprotected overnight (4° C in 30% sucrose 0.1 M PB), frozen in isopentane (-45° C) and stored (-80° C) until cutting of free-floating thick sections (25 µm) on a freezing-microtome. The remaining mice from each group were anesthetized with pentobarbital (80 mg/kg i.p.) and perfused transcardially with a 4% PFA solution. The brains were prepared as above, except that one hemisphere was processed for parafﬁn sectioning (5 µm-thick).

### Vascular reactivity

MCA segments (40–70 µm average intraluminal diameter) were cannulated, pressurized (60 mm Hg) and superfused with oxygenated Krebs solution (~37° C) for reactivity studies using online videomicroscopy [23]. Dilatory responses to acetylcholine (ACh; 10^-10^-10^-5^ M), calcitonin gene-related peptide (CGRP; 10^-10^-10^-6^ M) were tested on vessels pre-constricted submaximally with serotonin (5-HT; 2×10^-7^ M). Contractile responses to endothelin-1 (ET-1; 10^-10^-10^-6^ M), 5-HT (10^-10^-10^-6^ M) and to inhibition of nitric oxide synthase (NOS) with *N*ω-nitro-L-arginine (L-NNA; 10^-5^ M; 35min) were tested on vessels at basal tone. In the adult cohort, responses to 5-HT (10^-10^-10^-6^ M) and NO donor sodium nitroprusside (SNP; 10^-10^-10^-4^ M) were also tested. Changes in vessel diameter from either the basal or pre-constricted tone were expressed in percentage and plotted as a function of agonist concentration or time course of L-NNA incubation. Agonist efficacy and potency were compared using the maximal response (EAmax) and half maximal effective agonist concentration [EC50 value or p*D*_2_=-(log EC50)], respectively.

### ELISA measurement of Aβ_1-40_ and Aβ_1-42_

Soluble and insoluble Aβ_1-40_ and Aβ_1-42_ levels in cortex and hippocampus from hemibrains of adult and aged A/T mice were measured using an enzyme-linked immunosorbent assay (ELISA; BioSource International), as previously described [12]. Results are expressed as micrograms per gram (µg/g) of protein in the supernatant (soluble Aβ species) or formic acid fractions (insoluble Aβ species).

### Western blot

Proteins extracted from cerebral cortex and hippocampus (~20µg) were loaded onto a 15% Tris/tricine SDS PAGE and transferred to nitrocellulose membranes. Total soluble Aβ levels were detected with a mouse anti-Aβ1-16 antibody (6E10, 1:1000, Covance, Emeryville, CA, USA). Levels of proteins involved in vascular function and structure were also assessed in pial vessel extracts. Proteins (12-15 µg) loaded onto 10% SDS PAGE were probed with the following antibodies: goat anti-connective tissue growth factor (CTGF; 1:400; Abcam, San Francisco, CA, USA), -matrix metalloproteinase 9 (MMP9; 1:2000; Millipore, Temecula, CA, USA), -cyclooxygenase-2 (COX-2; 1:200; Cayman, Ann Arbor, MI, USA), rabbit anti-vascular endothelial growth factor (VEGF; 1:500; Santa Cruz Biotechnology, Santa Cruz, CA, USA), or -actin (1:8000; Santa Cruz Biotechnology A5441, Santa Cruz, CA, USA). Membranes were incubated (1h) with horseradish peroxidase-conjugated secondary antibodies (1:2000; Jackson ImmunoResearch, West Grove, PA, USA), and visualized with Enhanced ChemiLuminescence (ECL Plus kit; Amersham, Baie d’Urfé, QC, Canada) using phosphorImager (Scanner STORM 860; GE Healthcare, Baie d’Urfé, QC, Canada). Quantification was performed with ImageQuant 5.0 (Molecular Dynamics, Sunnyvale, CA, USA).

### Histochemical and Immunohistochemical stainings

Mature, dense core Aβ plaques were stained in 25µm-thick sections with 1% thioflavin S (8min). In similar sections, activated astrocytes and microglial cells were respectively immunodetected with a rabbit anti-GFAP antibody (1:1000; DAKO) or anti-ionized calcium binding adaptor molecule 1 (Iba-1; 1:300, Wako Chemicals, VA, USA), followed by a donkey anti-rabbit Cy2-conjugated secondary antibody (1:400; Jackson ImmunoResearch). Dewaxed thin paraffin sections (5 µm) were stained with 6E10 antibody to quantify total Aβ plaque load (1:2000). Sections were observed under a Leitz Aristoplan light microscope equipped with epifluorescence using an FITC filter (Leica). Pictures were taken with a Nikon digital camera (Coolpix 4500), and the percent area occupied by GFAP-, thioflavin S- and 6E10-positive immunostaining in cortex and/or hippocampus were quantified with MetaMorph 6.1r3 program (Universal Imaging).

### Statistical analysis

Data are expressed as means ± SEM and were analyzed by two-way ANOVA with genotype and treatment as factors or repeated measures ANOVA, followed by Newman-Keuls post-hoc multiple comparison test (Statistica for Academia, Tulsa, OK, USA). P values were reported if the interaction or at least one factor was significant. Two-group comparisons were analyzed by Student’s *t* test (GraphPad Prism 4, San Diego, CA, USA). A *p* < 0.05 was significant.

## Results

### Pioglitazone normalized neurovascular and neurometabolic coupling responses

**Figure 1:**
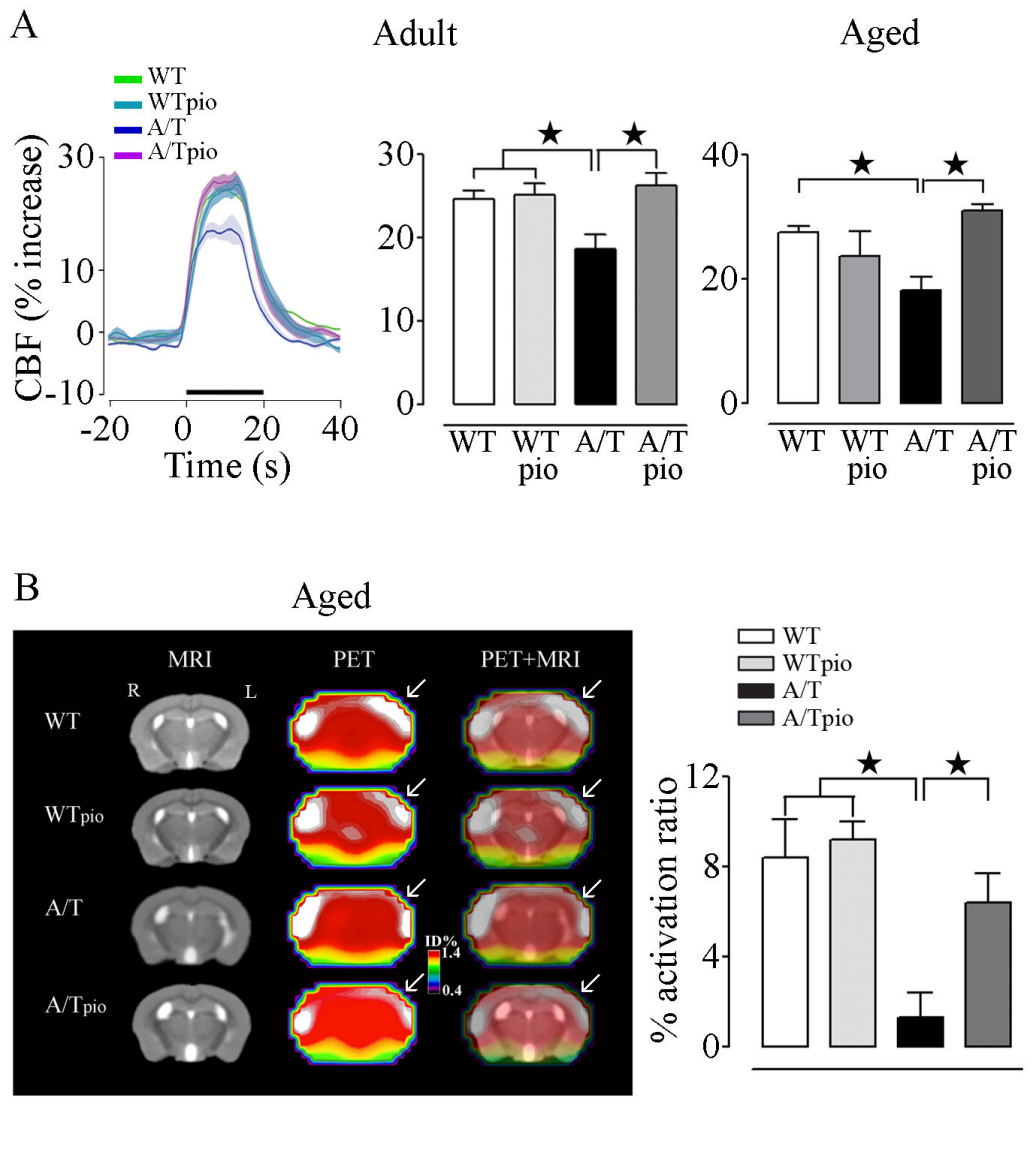
Pioglitazone (pio) restored stimulus-evoked CBF and CGU in A/T mice. ***A***, The impaired hyperemic response to whisker stimulation in adult and aged A/T mice was rescued by pioglitazone compared to age-matched WT controls, as measured by LDF (n=4 mice/group) (traces of adult mice shown, green: WT; turquoise: WTpio; violet: A/T and pink: A/Tpio). Values represent the percent increase in CBF induced by whisker stimulation relative to baseline. ***B***, Pioglitazone also improved the decreased CGU response to whisker stimulation in the somatosensory cortex of aged A/T compared to WT mice (n=3-6 mice/group). The % activation ratio denotes the percentage of corrected standard uptake value (SUV) in the activated contralateral (left side (L)) vs. ipsilateral (right side (R)) somatosensory cortex. Error bars represent SEM. ★p<0.05, ★★p<0.01, ★★★p<0.001 using two-way ANOVA followed by Newman-Keuls post-hoc test.

The CBF response to increased neuronal activity induced by whisker stimulation was significantly reduced in adult (18.7±1.7%; p<0.01) and aged (18.3±1.2%; p<0.01) A/T mice compared to age-matched WT controls (adult: 24.6±2.0% and aged: 27.6±1.0%), as shown by the laser Doppler flowmetry curves (adult cohort) and peak of CBF responses in cohorts of both ages (Figure 1A). Similarly, in aged A/T mice, the selective increase in CGU induced by whisker stimulation in the contralateral somatosensory cortex was significantly less than in WT littermates (% activation ratio: 1.3 ± 1.1 vs 8.4 ± 1.7 respectively; *p* < 0.05) (Figure 1B). Pioglitazone normalized the evoked CBF response to increased neuronal activity in both adult and aged A/T mice to levels comparable to their control counterparts (Figure 1A). The CGU response was also significantly improved by pioglitazone in aged A/T mice (A/Tpio: 6.4 ± 1.3; Figure 1B). Treatment had no effect on WT controls for both responses.

### Pioglitazone did not rescue spatial learning and memory – target quadrant

**Figure 2:**
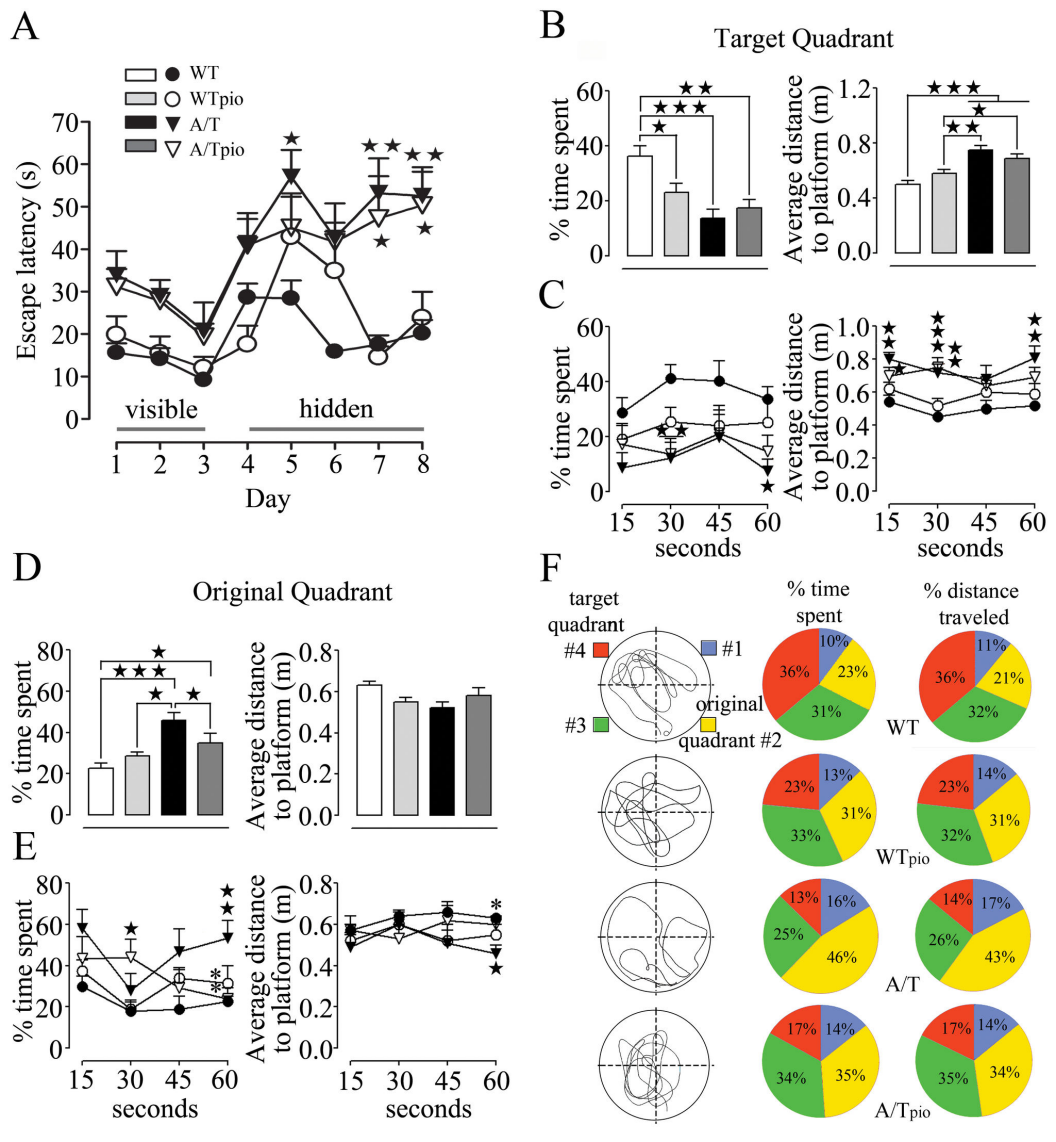
Effects of pioglitazone (pio) on spatial learning and memory in adult A/T mice. ***A***, A/T mice (▼) displayed impaired learning during hidden-platform testing compared to aged-matched wild-type (WT) littermates (●) in the Morris watermaze. These deficits were not due to visual or motor disabilities as all groups had comparable abilities to find the visible platform (days 1-3). Pioglitazone did not improve the deficit in A/T mice (▽) and did not affect the performance of WT controls in the hidden platform training (days 4-8). (○).B, A/T mice, treated (▽) and non-treated (▼) with pioglitazone displayed significant deficit in memory retention as assessed during the probe trial in the target quadrant. **C**, When the probe trial was subdivided in 15s segments for better understanding of the pattern of behavior throughout the 60s-long probe trial in target quadrant, pioglitazone did not exert any benefit in A/T mice. ***D***, A/T mice showed an inclination for the original quadrant during the probe trial, as shown by the increased time spent in this quadrant and smaller average distance to the platform location. ***E***, Patterns of behaviour in original quadrant segmented in 4 slices of 15s each during the probe trial showed that pioglitazone had a positive effect in A/T mice (▽) in increasing percent time spent and average distance to platform during in the last quarter of the probe trial. ***F***, Pie-representation of typical swimming patterns of % time spent and % distance traveled in target quadrant (red, #4) compared to the original quadrant (yellow, #2) during the probe trial. The drawings (on the left) are representations of single mouse swimming pattern best matching the average of of each group, as displayed by the pie patterns (on the right). Error bars represent SEM (n=6-9 mice/group). ★p<0.05 ★★, ** p<0.01, ★★★p<0.001 when compared to untreated WT controls (★) or A/T mice (*) using two-way ANOVA and repeated measures ANOVA followed by Newman-Keuls post-hoc test.

Adult and aged A/T mice displayed significantly increased escape latencies in finding the hidden platform compared to treated and untreated WT mice (Figure 2A). A/T mice also had a decreased inclination for the target quadrant during the probe trial, as shown for the adult cohort by the reduced time spent in this quadrant (#4) and greater average distance to the platform location (Figure 2B). These changes are indicative of impaired spatial learning and memory compared to their age-matched WT controls. These deficits could not be explained by visual or motor disabilities as all groups had comparable swim speeds (data not shown) and similar abilities to find the visible platform (days 1-3, Figure 2A). Further analysis of the search patterns in slices of 15s (Figure 2C) revealed that WT controls focused on the target quadrant throughout the probe trial with minimal navigation elsewhere (including in the original quadrant 2) (Figure 2B), as also depicted by their representative swim patterns (Figure 2F). In contrast, A/T mice navigated less in the target quadrant as the percent of time spent (Figure 2B) or distance traveled (A/T 13.8±3.3% vs. WT 36.2±3.8%, p<0.01) in the target quadrant were significantly less than in WT controls. Average distance to the platform was also larger in A/T mice for the majority of the 15s segments (Figure 2C). Pioglitazone treatment did not improve performance of A/T mice of either age group during the learning phase or probe trial, as shown in the adult cohort (Figure 2A–C). Notably, spatial memory in adult and aged pioglitazone-treated WT mice was slightly, but significantly, deteriorated (p<0.05, Figure 2), an effect not seen in previous WT cohorts after shorter [12] or longer [24] treatment duration.

### Pioglitazone and cognitive flexibility – original quadrant

In contrast to WT controls, both adult and aged A/T mice targeted the original quadrant (#2) throughout the probe trial, as shown here in adult mice by both increased percent time spent (Figure 2D) and distance traveled (WT 20.5±2.8% vs. A/T 42.6±4.5, p<0.01) in this quadrant. This persistence for the original platform location was apparent throughout most of the probe trial and was significant in the last 15s segmentation analysis for all parameters assessed including average distance from platform, percent time spent (Figure 2E), and distance travelled (WT 20.4±2.9% vs. A/T 54.7±8.8%, p<0.01). The preference of A/T mice for the original quadrant was obvious compared to all other quadrants, including the target quadrant (Figure 2F).

In adult, but not aged (data not shown), A/T mice pioglitazone had a positive effect in decreasing their time spent in the original quadrant and the average distance to platform (Figure 2D), specifically in the last quarter of the probe trial (Figure 2E). Similarly, the pioglitazone-treated adult A/T spent significantly less time (Figure 2E) and traveled less distance (A/T 42.6±4.5% vs. A/Tpio 24.5±5.2%, p<0.01) in the original quadrant during the last 15s-segment relative to untreated A/T mice. However, although their search towards the target quadrant was slightly improved, it was not complete since pioglitazone-treated mice predominantly explored in quadrant 3, as seen by their swimming patterns (Figure 2F). Hence, pioglitazone allowed for the treated-A/T mice to unlearn the original location of the visible platform without granting them the capacity to remember the new location of the hidden platform.

### Pioglitazone effects on brain vessel reactivity and fibrosis

**Figure 3:**
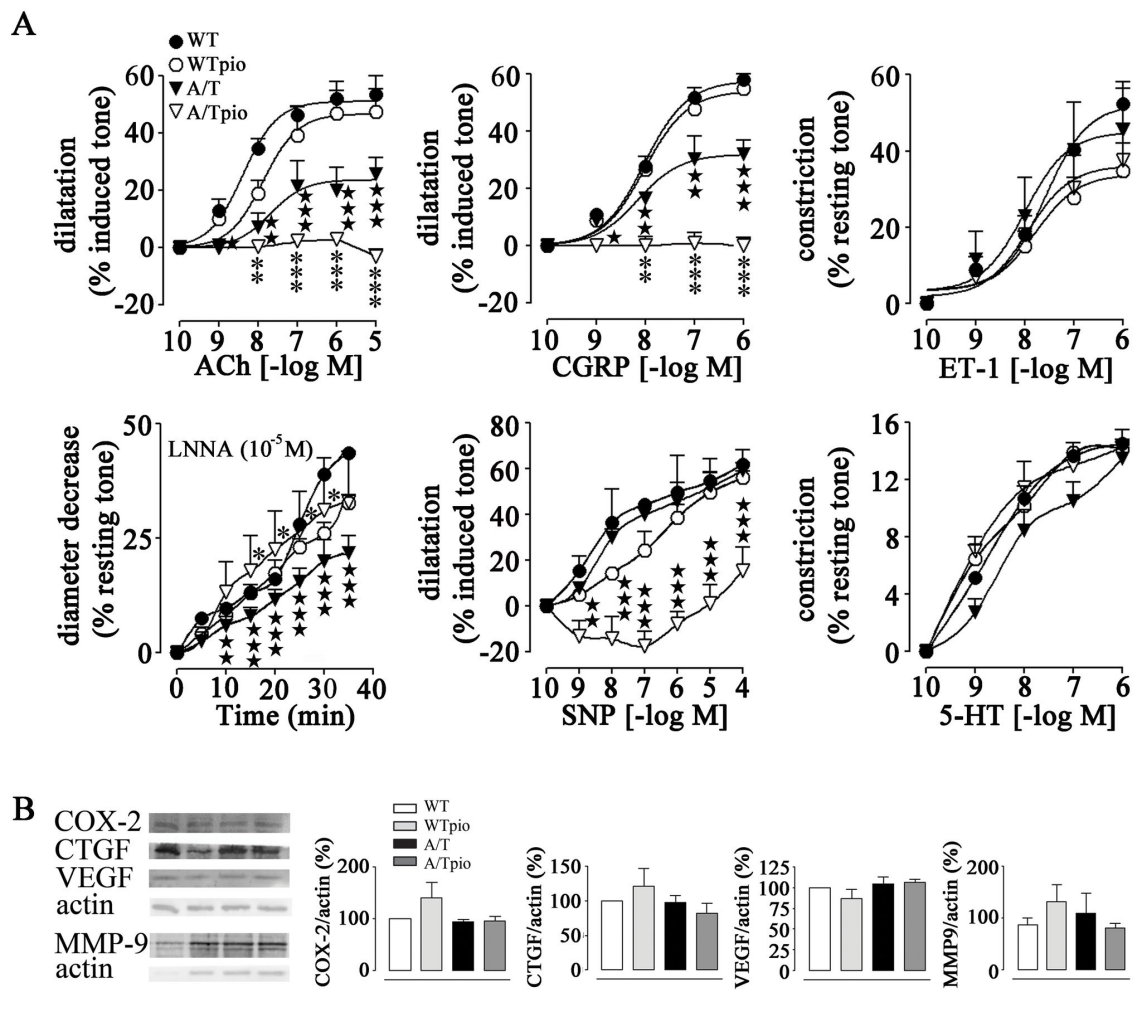
Effects of pioglitazone (pio) on cerebrovascular reactivity and protein alterations in adult A/T mice. ***A***, The impaired cerebrovascular dilatations to acetylcholine (ACh) and calcitonin gene-related peptide (CGRP) in A/T mice (▼) were aggravated or reversed to weak constrictions by pioglitazone (▽). This was accompanied by a full recovery of baseline NO synthesis in adult A/T mice measured during NOS inhibition (L-NNA, 10^-5^M). Dilatations induced by the NO donor SNP were unimpaired in A/T mice, but were reversed to small constrictions or shifted to the right at high SNP concentrations in pioglitazone-treated A/T mice. Contractile responses to 5-HT and ET-1 were unaltered in A/T mice and pioglitazone had no detrimental effects. Error bars represent SEM (n=3-6 mice/group). ***B***, Vasodilator synthesizing enzyme COX-2 and proteins associated with vascular fibrosis VEGF, CTGF and MMP-9 were not significantly altered by genotype or treatment, as measured by Western blot in pial vessels of adult A/T mice relative to their treated counterparts and WT mice. Actin was used as a reference for loading (n=4 mice/group). Similar results were obtained in the aged cohort, but data were not illustrated for clarity purposes. Error bars represent SEM. ★p<0.05, ★★, ** p<0.01, ★★★, *** p<0.001 when compared to untreated WT controls (★) or A/T mice (*) using two-way ANOVA (part A) and repeated measures ANOVA (part B) followed by Newman-Keuls post-hoc test.

In agreement with our previous study [5], adult and aged A/T mice displayed significantly decreased dilatory responses to ACh and CGRP compared to their age-matched WT controls (Tables 1 and 2), as illustrated in the adult group (Figure 3A). Reduced diameter decrease during NOS inhibition with L-NNA was also observed in A/T mice of both age groups, confirming a diminished constitutive NO synthesis. Despite these alterations in vascular reactivity, receptor desensitization was ruled out, as the agonist potencies at vascular receptors were strictly comparable between all groups (Tables 1 and 2). Arterial relaxation to the NO donor SNP and the constriction induced by ET-1 were normal in A/T mice, indicative of preserved contractile capacity, as also supported by the unaltered pre-constriction response to 5-HT (tested in the adult cohort only) (Figure 3A). Pioglitazone treatment partially (aged) or fully (adult) restored constitutive NO synthesis in A/T mice (Figure 3A, Tables 1, 2), and did not alter the contractile responses to 5-HT and ET-1. However, pioglitazone significantly deteriorated the dilatory responses to ACh and CGRP, resulting in no dilatation and weak contractile responses at high agonist concentrations (Figure 3A). Similarly, the NO donor SNP elicited vasoconstrictions at low doses that reverted to dilatations at higher concentrations (Figure 3A). These worsened responses were observed in both adult (Figure 3A, Table 1) and aged (Table 2) pioglitazone-treated A/T mice, although they were less pronounced in the adult cohort. Pioglitazone did not alter the cerebrovascular reactivity of WT mice (Figure 3A). The cerebrovascular levels of the prostaglandin-synthesizing enzyme COX-2, a vasodilator synthesizing enzyme (Figure 3B) [25] and of the pro-fibrotic proteins CTGF, VEGF or MMP9 [26,27] were not altered by genotype or treatment, as shown in the adult cohort (Figure 3B). These findings suggest that dysfunctions are not primarily caused by structural changes of the vessel wall.

**Table 1:** Effects of pioglitazone on cerebrovascular responses of adult A/T mice.

**Agonist**		**WT**	**WTpio**	**A/T**	**A/Tpio**
ACh	E_Amax_	51.2 ± 2.5	46.7 ± 4.0	23.7±3.6^★★★^	- 3.1± 3.6^★★★,^***
	pD_2_	8.4 ± 0.2	7.9 ± 0.2	7.7 ± 0.4	ND
CGRP	E_Amax_	57.6 ± 2.0	54.0 ± 3.6	31.9 ± 3.2^★★★^	- 0.4 ± 2.0^★★★,^***
	pD_2_	8.0 ± 0.1	8.0 ± 0.2	8.2 ± 0.2	ND
SNP	E_Amax_	62.0 ± 6.1	56.1 ± 3.0	59.5 ± 3.2	15.5 ± 10.1
5-HT	E_Amax_	14.6 ± 0.2	14.2 ± 0.5	13.5 ± 1.3	14.3 ± 1.2
ET-1	E_Amax_	52.3 ± 2.4	33.8 ± 4.1	44.9 ± 7.5	36.1 ± 5.0
	pD_2_	7.6 ± 0.1	7.8 ± 0.3	8.0 ± 0.5	7.9 ± 0.4
L-NNA	E_Amax_	43.7 ± 0.1	32.7 ± 1.9	21.8 ± 3.7^★★★^	32.8 ± 11.1*

Data are means ± SEM (n=3-6 mice per group) and are expressed as the agonist maximal response (EAmax) or potency (pD_2_, - [logEC_50_]). EAmax is the percent maximal dilatations to ACh, CGRP and SNP or the percent maximal diameter decreases to ET-1, 5-HT or after 35min inhibition with 10^-5^M L-NNA. ND: not determined. ★★★ p<0.001 when compared to untreated WT controls, *p<0.05 and ***p<0.001 when compared to untreated A/T mice by two way ANOVA followed by Newman-Keuls post-hoc multiple comparison test.

**Table 2:** Effects of pioglitazone on cerebrovascular responses of aged A/T mice.

**Agonist**		**WT**	**WTpio**	**A/T**	**A/Tpio**
ACh	E_Amax_	52.7 ± 2.0	54.5 ± 2.4	26.0±1.3^★★★^	16.0 ± 2.2^★★★^,***
	pD_2_	8.4 ± 0.1	8.3 ± 0.1	8.0 ± 0.1	ND
CGRP	E_Amax_	54.1 ± 1.6	55.6 ± 3.0	25.3 ± 1.4^★★★^	9.9 ± 2.1^★★★^,***
	pD_2_	8.8 ± 0.1	8.4 ± 0.1	8.3 ± 0.1	ND
ET-1	E_Amax_	58.3 ± 5.0	42.7 ± 3.6	53.4 ± 3.8	45.7 ± 4.0
	pD_2_	8.2 ± 0.2	8.3 ± 0.3	8.4 ± 0.2	8.2 ± 0.2
L-NNA	E_Amax_	52.3 ± 0.5	53.5 ± 5.1	15.1 ± 1.0^★★★^	28.7 ± 6.3^★★^

Data are means ± SEM (n=3-4 mice per group) and are expressed as the agonist maximal response (EAmax) or potency (pD_2_, - [logEC_50_]). EAmax is the percent maximal dilatations to ACh and CGRP or the percent maximal diameter decrease to ET-1 or after 35min inhibition with 10^-5^M L-NNA. ND: not determined. ★★ p<0.01, ★★★ p<0.001 when compared to untreated WT controls, and ***p<0.001 when compared to A/T mice by two way ANOVA followed by Newman-Keuls post-hoc multiple comparison test.

### Pioglitazone and amyloidosis

**Figure 4:**
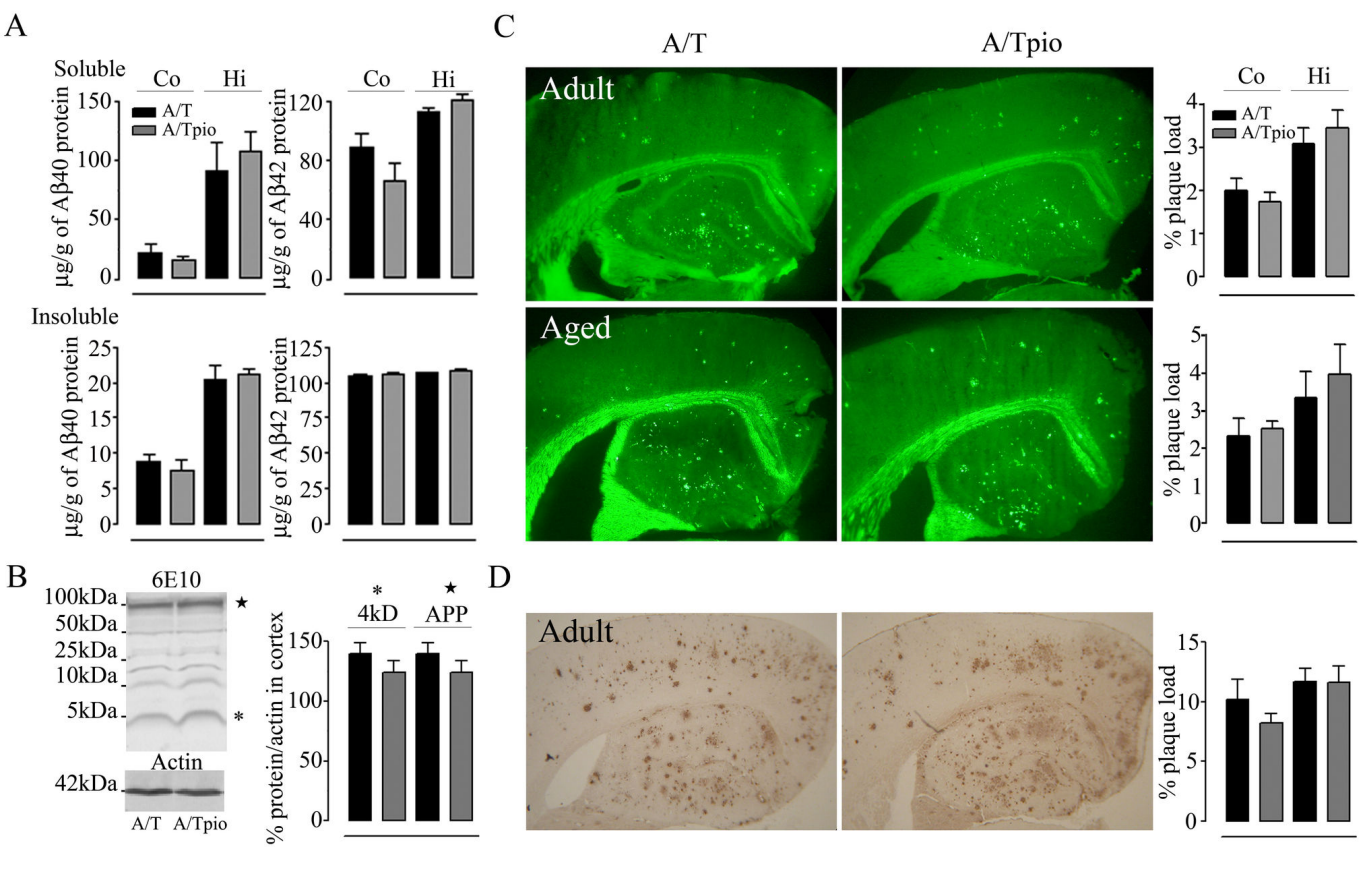
Pioglitazone (pio) had no effect on amyloidosis in A/T mice. ***A***, Levels of soluble and insoluble Aβ_1-40_ and Aβ_1-42_ in adult A/T mice, as assayed in cortex and hippocampus by ELISA, were comparable between pioglitazone-treated- and untreated A/T mice. ***B***, Western blot analysis with 6E10 antibody confirmed no effect of pioglitazone on soluble Aβ species in cortex of adult A/T mice. On the gel, (★) represents the APP band and (*), the 4kDa band of monomeric Aβ including Aβ_1-40_ and Aβ_1-42_, as quantified in the bar graph. ***C***, Thioflavin S-stained dense-core Aβ plaque load in cortex and hippocampus were unaltered by pioglitazone in adult A/T mice. ***D***, Similarly, 6E10-immunostaining of diffuse and dense-core Aβ plaques in 5µm-thick paraffin sections showed no effect of pioglitazone on cortical and hippocampal Aβ plaque load in adult A/T mice. Error bars represent SEM. ★p<0.05, ★★p<0.01, ★★★p<0.001 for comparison to A/T mice using two-way ANOVA followed by Newman-Keuls post-hoc test (n=4 mice/group).

Pioglitazone had no effect on soluble and insoluble Aβ_1-40_ and Aβ_1-42_ levels measured by ELISA in cortex and hippocampus of adult (Figure 4A) and aged (data not shown) A/T mice. High total soluble Aβ levels were also detected in cortex and hippocampus of adult and aged A/T mice by Western blot and, similarly, pioglitazone had no benefit at either age, as shown in cortex of adult A/T mice (Figure 4B). Plaque load of thioflavin S-positive dense core plaques was unaltered by pioglitazone in cortex and hippocampus from A/T mice irrespective of age and length of treatment (Figure 4C). This was further confirmed by the unaltered load of diffuse and dense core 6E10-immunopositive Aβ plaques measured in thin sections (Figure 4D). Together, these results demonstrate that pioglitazone does not counter the amyloid pathology in A/T mice, in line with previous studies in singly APP mice [10,12].

### Pioglitazone and neuroinflammation

**Figure 5:**
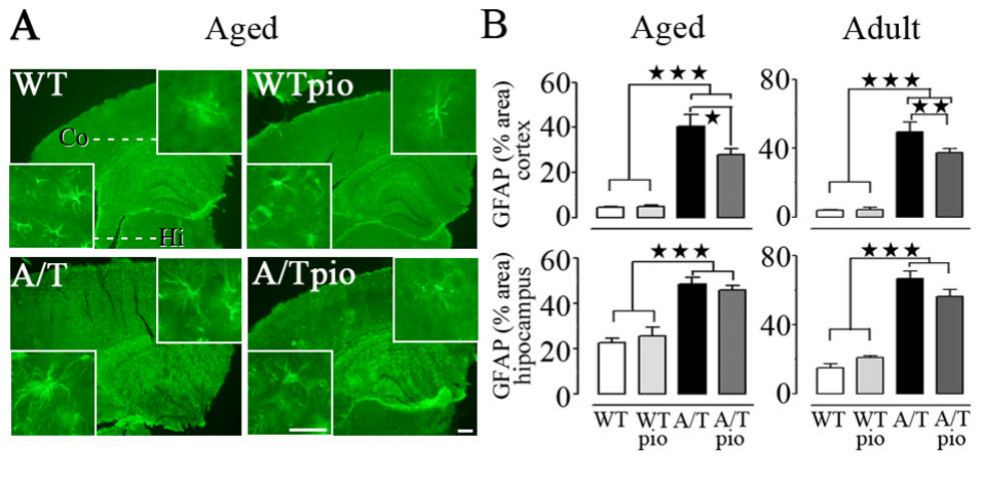
Pioglitazone (pio) reduced cortical astroglial activation. ***A***, In the cortex (but not in the hippocampus) of aged A/T mice, pioglitazone significantly reduced the increase in GFAP-immunoreactive (IR) surface area relative to WT controls (n=4 mice/group). ***B***, GFAP analysis of both the aged and adult groups rendered the same results of a selective cortical effect of pioglitazone on astroglial activation. Scale bar = 300 µm and 100 µm (insert). Error bars represent SEM. ★p<0.05, ★★p<0.01, ★★★p<0.001 using two-way ANOVA followed by Newman-Keuls post-hoc test.

**Figure 6:**
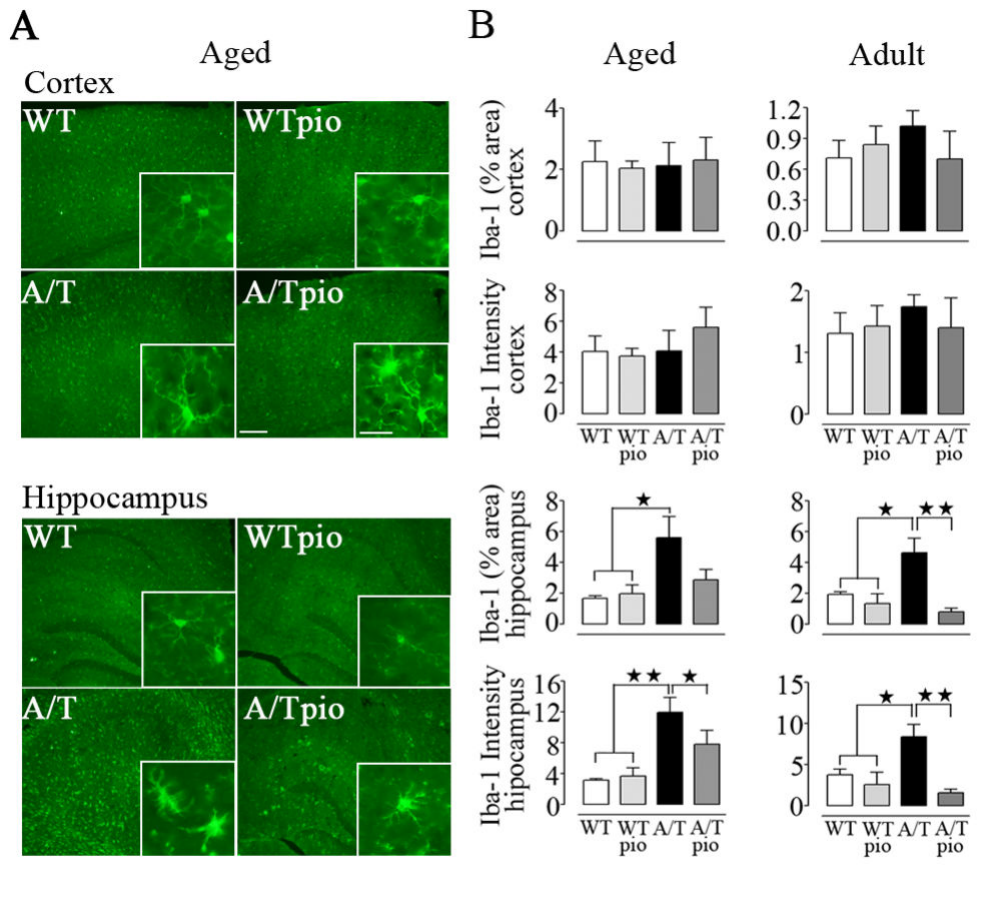
Pioglitazone (pio) reduced hippocampal microglial activation. ***A***, Pioglitazone significantly reduced the increased surface area of Iba-1-immunolabelled microglial cells in the hippocampus of both aged and adult A/T mice compared to WT controls, as shown in the aged group. ***B***, Both the area occupied by and staining intensity of Iba-1-positive microglial cells in the hippocampus were reduced by pioglitazone at both ages. No effect was seen in the cortex (n=4 mice/group). Scale bar = 100 µm and 25 µm (insert). Error bars represent SEM. ★p<0.05, ★★p<0.01 using two-way ANOVA followed by Newman-Keuls post-hoc test.

Both adult and aged A/T mice exhibited a significant increase in GFAP-positive area in cortex and hippocampus compared to their age-matched WT controls (p<0.001) (adult cohort, Figure 5A, B). In both cohorts, pioglitazone significantly reduced astroglial activation only in the cortex where activation was not as severe as in the hippocampus (Figure 5B). Microglia were also activated as evidenced by the percent area occupied by and intensity of Iba-1-immunstained microglial cells in the hippocampus but not in the cortex of A/T mice of both ages compared WT controls (Figure 6A, B). Pioglitazone exerted significant protective effects in minimizing microglial activation in the hippocampus of A/T mice (Figure 6 A, B).

## Discussion

This study demonstrates positive effects of pioglitazone on neurometabolic and neurovascular coupling responses, and on neuroinflammation in a mouse model that recapitulates the AD-related Aβ pathology, cognitive deficits and cerebrovascular alterations. A modest improvement in reversal learning, suggesting improved cognitive flexibility (i.e., less perseveration) was also observed in the adult, but not in the aged cohort, notwithstanding persistent memory deficits and amyloidosis in both age groups. However, the contractile effect of pioglitazone on brain vessels of A/T mice pointed to possible detrimental effects in AD patients with vascular diseases.

### Pioglitazone on neurometabolic and neurovascular coupling responses

Early decreases in resting CGU have been reported in the parietotemporal and posterior cingulate cortex in AD patients [28–30] and in various cortical areas in APP mice [31] using FDG-PET and autoradiography, respectively. Altered neurometabolic coupling in response to increased neuronal activity has also been reported in AD patients [32], APP mice [12] and aged A/T mice [5]. The finding that pioglitazone improved CGU during activation of the somatosensory cortex in the combined Aβ- and TGF-β1-burdened brain of aged A/T mice underscored the high efficacy of pioglitazone in restoring brain glucose metabolism. *In vivo* experiments in healthy rat brain showed that glucose preferentially fuels astrocytes during neuronal activity even though neurons have greater metabolic needs [33]. Astrocytes then supply neurons with glycolytic products, particularly lactate, for use in oxidative metabolism [34]. Hence, our FDG-PET results may reflect both restored astrocytic glucose metabolic activity and neuronal oxidative metabolism by pioglitazone, similar to what has been demonstrated *in vitro* [35,36]. In support of the normalized astrocytic function by pioglitazone is our finding of reduced cortical GFAP-immunostaining intensity, a marker of increased astrocytic-reactive inflammatory response [37]. An achieved astroglial homeostasis could also explain the normalization of the functional hyperemic response that requires the concerted action of neurons, astrocytes and vascular cells [38,39]. Particularly, PPARγ activation may have facilitated the ability of astrocytes to synthesize arachidonic acid derivatives such as vasodilatory epoxyeicosatrienoic acids (EETs) that mediate a large part of the neurovascular coupling response to whisker stimulation [25,40], a response severely affected in both APP and A/T mice [5,41]. However, the pioglitazone-mediated anti-inflammatory properties evidenced by the silencing of cortical astrocytes and hippocampal microglial cells that are thought to participate in the clearance of aggregated Aβ species [42], did not impact on either soluble Aβ species or Aβ plaque load. This finding is in line with some studies in APP mice [10,12], but not with others that reported decreased plaque load or Aβ processing [11,43] following pioglitazone treatment.

### Pioglitazone and spatial memory

Despite the beneficial effects of pioglitazone highlighted above, spatial learning and memory in adult or aged A/T mice was not improved, as in our previous findings in aged APP mice [12]. This may indicate that, despite hypoperfusion [44,45] and hypometabolism [32] being good correlates of memory decline in AD, local recovery of CBF and CGU responses in the somatosensory cortex of pioglitazone-treated A/T mice are not good indicators of changes in hippocampus-based mnemonic function. Based on the heterogeneity in brain glucose metabolism, pioglitazone may have failed to normalize the metabolic deficit in the limbic system in A/T mice, as previously reported in TGF mice [46]. Indeed, in contrast to its beneficial effects in cortex, pioglitazone did not reduce astrocytic activation in the hippocampus. Since astrocytes are important in maintaining hippocampal homeostasis that is critical for memory formation [47], we suggest that their persistent activated state might contribute to the lack of memory rescue in treated A/T mice. Future investigations should ideally evaluate the evoked CGU or CBF responses in the hippocampus, and use hippocampus-specific stimulation paradigms that would better correlate with memory performances.

In a study on a small population of mild AD patients with type II diabetes treated with pioglitazone, resting CBF improvement in the parietal lobe – including the post-central gyrus (encompassing the somatosensory cortex) – was accompanied by cognitive improvement [8]. This beneficial effect was also concurrent with an increase in insulin-mediated glucose uptake [8], a known mechanism of action for PPARγ [48]. However, aside from one additional study showing cognitive improvement in diabetic AD patients with pioglitazone [49], all other clinical trials of AD patients failed to demonstrate benefit [7]. It is thus tempting to speculate that pioglitazone may confer cognitive protection only in AD patients with diabetes.

### Pioglitazone and cognitive flexibility

Our data demonstrated that A/T mice exhibited a moderate preference for the original quadrant during the probe trial, as opposed to the target quadrant. Our protocol consisted of a single visible-to-hidden platform relocation and, as such, was less robust than the memory flexibility test of Chen and colleagues who used a series of hidden-platform relocation trials for the measurement of hippocampal-based learning deficits [19]. Nevertheless, our data show that pioglitazone exerted beneficial effects on the impaired reversal learning of A/T mice. Pioglitazone facilitated their ability to “switch” from a perseverative (and incorrect) strategy in exploring in the original quadrant, to new strategies in relocating the hidden platform of the target quadrant, and this, only in the adult cohort. These positive results point to age and treatment duration being both important elements in this beneficial effect of pioglitazone. Interestingly, rosiglitazone, another PPARγ agonist, similarly enhanced cognitive flexibility in APP/PS1 mice [50]. Hence, this new therapeutic potential uncovered for pioglitazone in adult A/T mice may bear clinical significance for AD patients in light of their reported impaired reversal learning [51]. Yet, a therapeutic benefit may be highly dependent on the stage of the disease and time of treatment initiation.

### Pioglitazone and cerebrovascular reactivity

Recovery of cerebrovascular reactivity with pioglitazone was anticipated based on its proven benefit in aged APP [12] and TGF [14] mice. Whereas vasoconstriction to ET-1 and 5-HT remained unaffected, indicative of preserved smooth muscle integrity, the impaired ACh- and CGRP-mediated dilations in A/T brain vessels were aggravated or reversed to small constrictions after pioglitazone treatment. This worsening behavior might relate to the ability of pioglitazone to directly elicit concentration-dependent dilations through endothelium-mediated NO release and gyanylyl cyclase activation, as evidenced in retinal arteries [52] or aorta [53]. Additionally, the ability of pioglitazone to inhibit ATP-sensitive K^+^ (KATP) channels, as shown in the coronary circulation [54] may explain its adverse effects on CGRP-induced dilations that are largely mediated by KATP channels [55–57]. Although less involved in CGRP-mediated relaxation [58], the activation of smooth muscle voltage gated K^+^ (Kv) channels by pioglitazone [52] may also have contributed to its worsening effect. It thus appears that chronic pioglitazone treatment in A/T mice hindered the NO signaling cascade or K^+^ channel activation required for ACh- and CGRP-mediated dilatations [55–57]. This possibility is reinforced by the failure of the NO donor SNP to induce dilation, pointing to mechanisms downstream of NO production. The finding that baseline NO production was not altered by pioglitazone further points to dysfunction in NO signaling. However, we cannot exclude that cerebral amyloid angiopathy (CAA), which is accrued in A/T mice compared to APP mice [59], contributed to the altered reactivity through other mechanisms [60]. Although less potent at inhibiting KATP channels than rosiglitazone [54], another PPARγ agonist recently prohibited from use in diabetic patients for its high-risk cardiovascular profile [61], our findings point to potential deleterious effects of pioglitazone on cerebral dilatory function.

## Conclusion

Our results show that pioglitazone exerts beneficial effects in A/T mice with multiple AD hallmarks. Pioglitazone countered cortical neuroinflammation, neurometabolic and neurovascular coupling responses to increased neuronal activity in sensory pathways, with an age-dependent improvement in reversal learning, pointing to benefits at several levels of the AD pathology and to potential use as a combined therapy. However, the A/T mouse model unmasked previously unrecognized, possibly deleterious, effect of chronic pioglitazone therapy on dilatory function, particularly those involving NO signaling. This finding may be relevant to AD patients with comorbid cardiovascular diseases despite the better cardiovascular safety profile of pioglitazone compared to rosiglitazone [62,63].
